# A Comparison of 1.5-Stage and Two-Stage Revisions for Prosthetic Joint Infection in Total Hip and Knee Arthroplasty: A Meta-Analysis of Outcomes

**DOI:** 10.7759/cureus.98180

**Published:** 2025-11-30

**Authors:** Abdelrahman Ibrahim, Khadija Khamdan, Salman Sadiq, Ahmed Lyeeq, Nikhil Narayanswamy, Abu Saeed

**Affiliations:** 1 Trauma and Orthopaedics, Princess Royal Hospital NHS Trust, Telford, GBR; 2 Trauma and Orthopaedics, University Hospitals of North Midlands NHS Trust, Stoke-on-Trent, GBR; 3 Trauma and Orthopaedics, Russells Hall Hospital, Dudley, GBR; 4 Trauma and Orthopaedics, Worcestershire Acute Hospitals Trust, Worcester, GBR

**Keywords:** 1.5-stage revision, meta-analysis, prosthetic joint infection (pji), tka (total knee arthoplasty), total hip arthroplasty (tha), two-stage revision arthroplasty

## Abstract

Prosthetic joint infection (PJI) is a devastating complication of total hip and knee arthroplasty. Whilst two-stage revision has long been considered the gold standard treatment, the 1.5-stage revision has emerged as a viable alternative. However, the optimal surgical strategy remains a subject of debate. The aim of this meta-analysis was to evaluate the comparative outcomes of 1.5-stage versus two-stage revision for PJI.

A systematic search of electronic data sources and bibliographic reference lists was conducted. All studies reporting comparative outcomes of 1.5-stage versus two-stage revision were included, and their risk of bias was assessed. Reinfection, failure of infection eradication, aseptic loosening, overall complications, readmission, and periprosthetic fracture were the evaluated outcome parameters.

All comparative studies reporting on patients who underwent either a 1.5-stage or a two-stage revision were included in the final analysis. The 1.5-stage revision was associated with a significantly lower rate of reinfection (odds ratio (OR): 0.62; 95% confidence interval (CI) 0.40-0.96, p = 0.03) but a significantly higher rate of aseptic loosening (OR: 6.12; 95% CI 1.09-34.22, p = 0.04) when compared with the two-stage revision. No significant difference was found in the rates of infection eradication (OR: 1.35; 95% CI 0.80-2.27, p = 0.26), overall complications, readmission, dislocations, or periprosthetic fracture between the two groups. A subgroup analysis for knee PJI was consistent with the main analysis for reinfection.

The meta-analysis of the best available evidence indicates that a 1.5-stage revision for PJI is associated with a lower rate of reinfection but a higher risk of aseptic loosening. High-quality randomized controlled trials are needed to definitively establish the optimal surgical strategy for managing PJI.

## Introduction and background

Periprosthetic joint infection (PJI) is a catastrophic complication following total joint arthroplasty (TJA), with an incidence of 1% to 2% [1-3]. Mortality after a PJI has been reported to be as high as 11% in total hip arthroplasty (THA) and 14.4% in total knee arthroplasty (TKA), which is comparable to common cancers [3,4]. As the volume of primary and revision TJA procedures is projected to grow substantially, PJI is becoming the leading cause for early revisions [5], placing a great burden on the healthcare system with annual costs expected to reach $1.85 billion by 2030 in the United States [6,7].

Two-stage revision arthroplasty has historically been considered the gold standard for treating chronic PJI [8,9]. Absolute indications for staged revision have historically included sinus formation and antibiotic-resistant polymicrobial organisms. However, this approach is associated with significant challenges, including high attrition and mortality rates, as well as mechanical complications of hip spacers such as dislocations and fractures [1,10]. Furthermore, the morbidity, cost implications, and substantial psychological impact on the patient are considerable [11,12].

A more novel technique described as the 1.5-stage revision has gained popularity in both North America and Europe more recently [13]. This technique most commonly involves the insertion of primary cemented arthroplasty components, which function as articulating spacers with the aim of potentially delaying the second stage indefinitely [14]. In the case of hip replacements, this involves a cemented femoral stem and a cemented polyethylene acetabular component. This is augmented with local antibiotic delivery to the soft tissues and medullary canals, which can be achieved through various methods. The theoretical advantages of a 1.5-stage include improved function with an articulating spacer, easier removal compared to revision components if required, preservation of bone stock, and cost benefits [11,15].

Although both the 1.5-stage and two-stage exchange are utilized, the ideal treatment for PJI remains controversial [9]. The current literature on the 1.5-stage approach is characterized by promising reports but is largely limited by small sample sizes and a paucity of direct comparative data against the traditional two-stage method [16]. Case series have shown the 1.5-stage to be associated with greater infection-free survival compared to the two-stage for both THA (94% vs. 83%, p = 0.048) and TKA (85.1% vs. 75.0%, p = 0.158) [17]. Nabet et al. also reported that the 1.5-stage revision had a significantly lower rate of complications when compared to the two-stage revision (8.8% vs. 31.3%, p < 0.001) [18].

Recent meta-analyses have begun [19] to address this gap, but the emergence of new studies [13,20] warrants an updated evidence synthesis. Furthermore, THA and TKA present unique challenges, and this is the first meta-analysis to assess outcomes specific to the hip and knee joints independently.

The primary aim of this meta-analysis was to compare the failure of infection eradication, reinfection, and complications between 1.5- and two-stage revisions for PJI. The secondary aim is to compare readmission, periprosthetic fracture, and aseptic loosening rates between the two groups. We will also perform a subgroup analysis for the THA and TKA groups.

## Review

Methods

The eligibility criteria, methodological framework, and investigated outcome parameters of this study were defined in advance and documented in a review protocol. The methodology followed and adhered to the standards set by the Preferred Reporting Items for Systematic Reviews and Meta-Analyses (PRISMA) guidelines [21].

Study Design

The inclusion criteria included all comparative observational studies comparing the outcomes of 1.5-stage versus two-stage revision in patients with periprosthetic infection in THA and/or TKA. The exclusion criteria included studies that did not directly compare 1.5- and two-stage revisions for any outcomes and studies with populations under 18 years of age. Articles in languages other than English were also excluded.

Eligibility Criteria

The primary population of interest included all adult patients (age > 18) of any gender undergoing revision surgery for a PJI. The intervention group consisted of patients undergoing a 1.5-stage revision, which was compared to a control group of patients undergoing a traditional two-stage revision.

Outcomes

The primary outcomes were defined as infection eradication, reinfection, and aseptic loosening. Secondary outcomes included overall complications, readmission, periprosthetic fracture, and dislocations.

Literature Search Strategy

A comprehensive search strategy was developed (Appendix) according to thesaurus headings, relevant search operators, and database-specific limits within MEDLINE, Web of Science, and EMBASE. Two authors independently carried out the literature search and evaluated clinical trial registries, including the International Standard Randomized Controlled Trial Number (ISRCTN) registry, ClinicalTrials.gov, and the WHO International Clinical Trials Registry Platform (ICTRP) to identify any ongoing or unpublished studies. Additionally, the reference lists of all included studies were screened for additional potentially eligible articles. The last literature search was carried out on August 5, 2025.

Selection of Studies

Two independent reviewers screened all titles and abstracts identified through the search. Where required, the full texts of relevant articles were obtained and carefully assessed against the predefined eligibility criteria of this review. Studies that met the inclusion criteria were included for the analysis. Any discrepancies arising during this selection phase were resolved through discussion between the two authors. However, if the disagreement persisted, a third author was consulted.

Data Extraction and Management

A standardized electronic data extraction sheet was developed in line with Cochrane guidance for intervention reviews. It was then pilot-tested using randomly selected articles and subsequently refined. Two independent reviewers extracted information on study-related data, baseline demographic and clinical characteristics of the study populations, and outcome data from each of the included studies. Any discrepancies encountered were resolved through discussion with a third author.

Assessment of Risk of Bias

The methodological quality and risk of bias of observational studies were assessed independently by two authors using the Risk Of Bias In Non-randomized Studies-of Interventions, Version 2 (ROBINS-I V2) assessment tool. ROBINS-I V2 is designed to assess the risk of bias in a specific result from a non-randomized study examining the effect of an intervention on an outcome [22]. An algorithm within the tool assesses multiple domains of bias to generate an overall judgment, categorizing the risk for a specific result as either 'low', 'unclear', or 'high'. Any discrepancies identified during this assessment were resolved by discussion between the two authors. If a consensus could not be reached, a third author was consulted for adjudication.

Summary Measures and Synthesis

For the evaluated dichotomous outcomes (e.g., failure of infection eradication and reinfection), the odds ratio (OR) with a 95% confidence interval (CI) was used as the summary measure. As all reported dichotomous outcomes were adverse events, an OR greater than 1 indicated a higher risk associated with the 1.5-stage revision group, thus favouring the two-stage revision group. Conversely, an OR less than 1 would favour the 1.5-stage revision group.

Data extracted from the included studies were initially entered by one reviewer into Review Manager 7.12.0 software (The Cochrane Collaboration, London, UK) for subsequent analysis [23]. The accuracy of this entered data was then independently verified by a second review author. Random-effects modelling was used for analysis. The results of the analysis for each outcome parameter were reported in a forest plot, along with 95% CIs. The extent of heterogeneity among the studies was assessed using the Cochran Q test (χ^2^). Inconsistency was quantified by calculating I² and was interpreted according to the following guide: 0%-25% suggesting possibly unimportant heterogeneity, 26%-75% indicating moderate heterogeneity, and 76%-100% representing high heterogeneity.

Furthermore, to investigate potential sources of heterogeneity and confirm the robustness of our findings, we planned to perform sensitivity analyses. This could include a specific analysis excluding studies with a particularly high risk of bias. Furthermore, the influence of each individual study on the overall effect size and heterogeneity was assessed by re-running the analysis, sequentially omitting one study at a time.

Results

**Figure 1:**
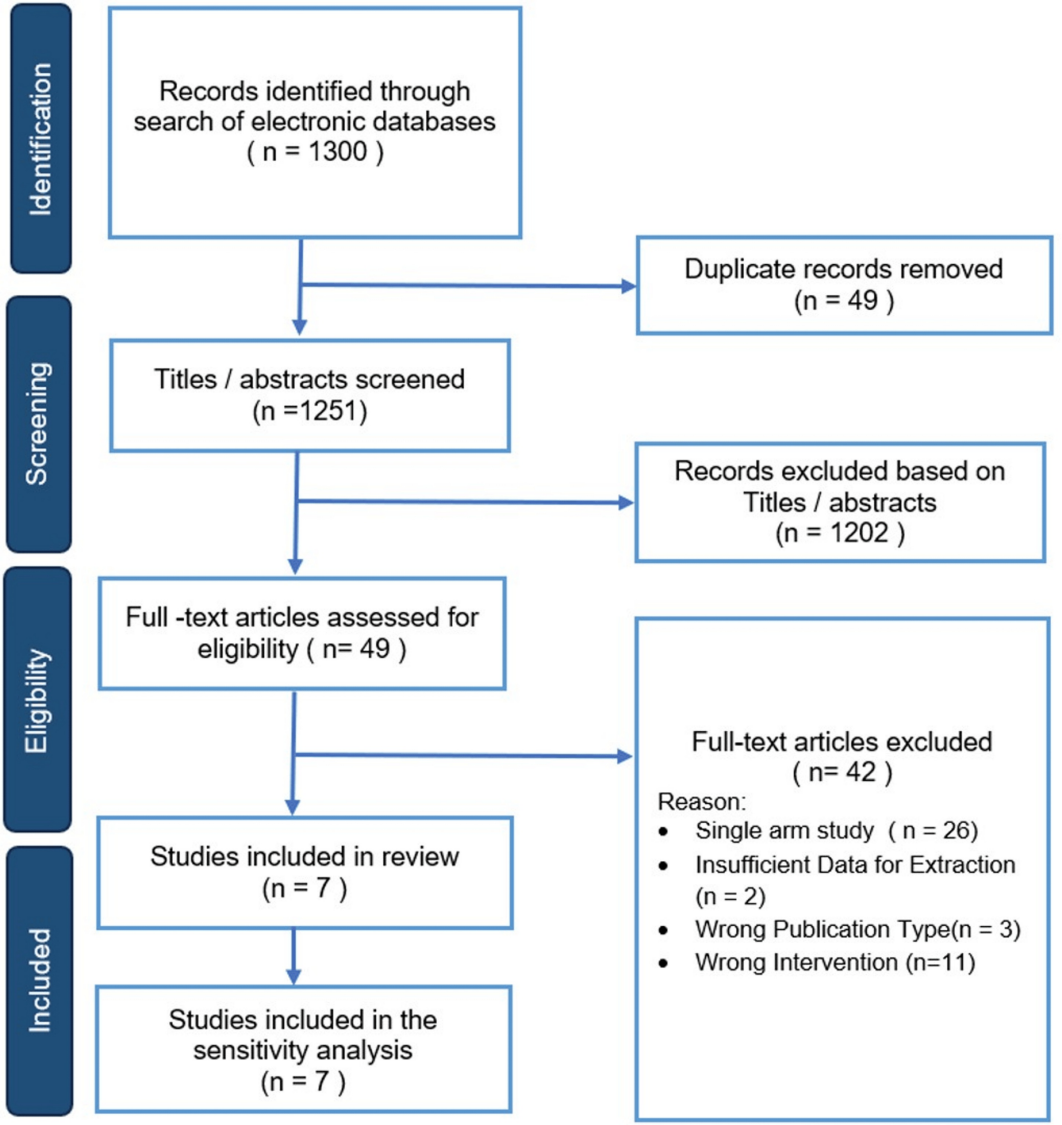
PRISMA flow diagram. PRISMA: Preferred Reporting Items for Systematic Reviews and Meta-Analyses

Following the literature search, 1,300 articles were identified. After the removal of 49 duplicates, 1,251 articles were screened. Of those, 49 articles were shortlisted for full-text assessment and potential inclusion. After careful evaluation of their full texts, 42 studies were excluded for the following reasons: 26 were single-arm studies, 11 had the wrong intervention, three had the wrong publication type, and two had insufficient data for extraction. Therefore, we included seven comparative studies, all of which were retrospective cohort studies, enrolling a total of 767 patients who underwent either 1.5-stage revision (n = 371) or two-stage revision (n = 396) (Figure 1).

Study and Baseline Characteristics

The baseline characteristics of the two groups were comparable. The weighted mean age was 64.0 years for the 1.5-stage group and 62.2 years for the two-stage group. The weighted mean percentage of women was 42.2% and 52.9%, respectively. Similarly, the weighted mean BMI was 31.87 kg/m² versus 33.08 kg/m², and the mean follow-up was 33.3 months versus 38.6 months, respectively. Detailed study-related data and baseline characteristics are presented in Table 1.

**Table 1:** Study-related and baseline characteristic data ¹The study by Zamora et al., 2020 [26] reported overall means for age, gender, and follow-up References: [13,17,18,20,24-26]

Author	Country	Study design	Total	1.5-stage revision (n=)	Two-stage revision (n=)	Mean age (years) 1.5- vs. two-stage	Sex (female %) 1.5- vs. two-stage	Mean BMI 1.5- vs. two-stage	Follow-up duration (months ) 1.5- vs. two-stage
Belay et al., 2023 [24]	USA	Retrospective cohort study	116	58	58	66 vs. 65	46.6% vs. 43.1%	34.0 vs. 33.6	32.2 vs. 28.1
Nabet et al., 2022 [18]	USA	Retrospective cohort study	162	114	48	64 vs. 62	43.0% vs. 58.3%	34 ± 10 vs. 37 ± 10	31.2 vs. 30.0
Nace et al., 2023 [17]	USA	Retrospective cohort study	123	54	69	61 vs. 59	31.5% vs. 49.3%	31 vs. 33	31.2 vs. 27.6
Siddiqi et al., 2023 [25]	USA	Retrospective cohort study	194	57	137	NR	47.4% vs. 64.2%	31.7 ± 7.3 vs. 33.4 ± 8.4	52.9 vs. 54.7
Villa et al., 2025 [13]	USA	Retrospective cohort study	73	43	30	65.7 vs. 63.4	44.2% vs. 33.3%	29.1 vs. 29.4	10.4 vs. 21.4
Wang et al., 2024 [20]	China	Retrospective cohort study	50	27	23	63 vs. 64	37.0% vs. 34.8%	24.80 vs. 26.72	44.4 vs. 42.0
Zamora et al., 2020 [26]	Canada	Retrospective cohort study	49	18	31	69¹	49.00%¹	NR	34 months¹

Surgical Methods

Details of the implants and fixation methods used in the included studies are outlined in Table 2. For knee arthroplasty, the 1.5-stage revisions consistently utilized cemented, primary TKA implants. For hip arthroplasty, the techniques were more varied, including fully cemented, hybrid, and cementless fixations with both primary and revision components. Information on the specific surgical methods for the two-stage revision groups was often not specified in the included studies.

**Table 2:** Implants and fixation methods in 1.5-stage vs. two-stage revisions References: [13,17,18,20,24-26] TKA: total knee arthroplasty

Study	Arthroplasty	Fixation method	Implant type
Belay et al., 2023 [24]	Knee	1.5-stage: cemented	1.5-stage: retained articulating spacer
		Two-stage: NR	Two-stage: NR
Nabet et al., 2022 [18]	Knee	1.5-stage: cemented	1.5-stage: primary TKA implants
		Two-stage: NR	Two-stage: NR
Nace et al., 2023 [17]	Hip	1.5-stage: cemented	1.5-stage: cemented femoral stem and a cemented standard or dual-mobility polyethylene liner
		Two-stage: NR	Two-stage: NR
Siddiqi et al., 2023 [25]	Knee	1.5-stage: cemented	1.5-stage: primary TKA implants (unconstrained posterior-stabilized)
		Two-stage: NR	Two-stage: NR
Villa et al., 2025 [13]	Hip	1.5-stage: hybrid fixation	1.5-stage: revision/complex primary components
		Two-stage: primarily cementless	Two-stage: revision components
Wang et al., 2024 [20]	Hip	1.5-stage: cemented	1.5-stage: primary arthroplasty components
		Two-stage: NR	Two-stage: NR
Zamora et al., 2020 [26]	Knee	1.5-stage: cemented	1.5-stage: primary TKA implants (posterior-stabilized)
		Two-stage: NR	Two-stage: NR

Methodological appraisal

**Figure 2:**
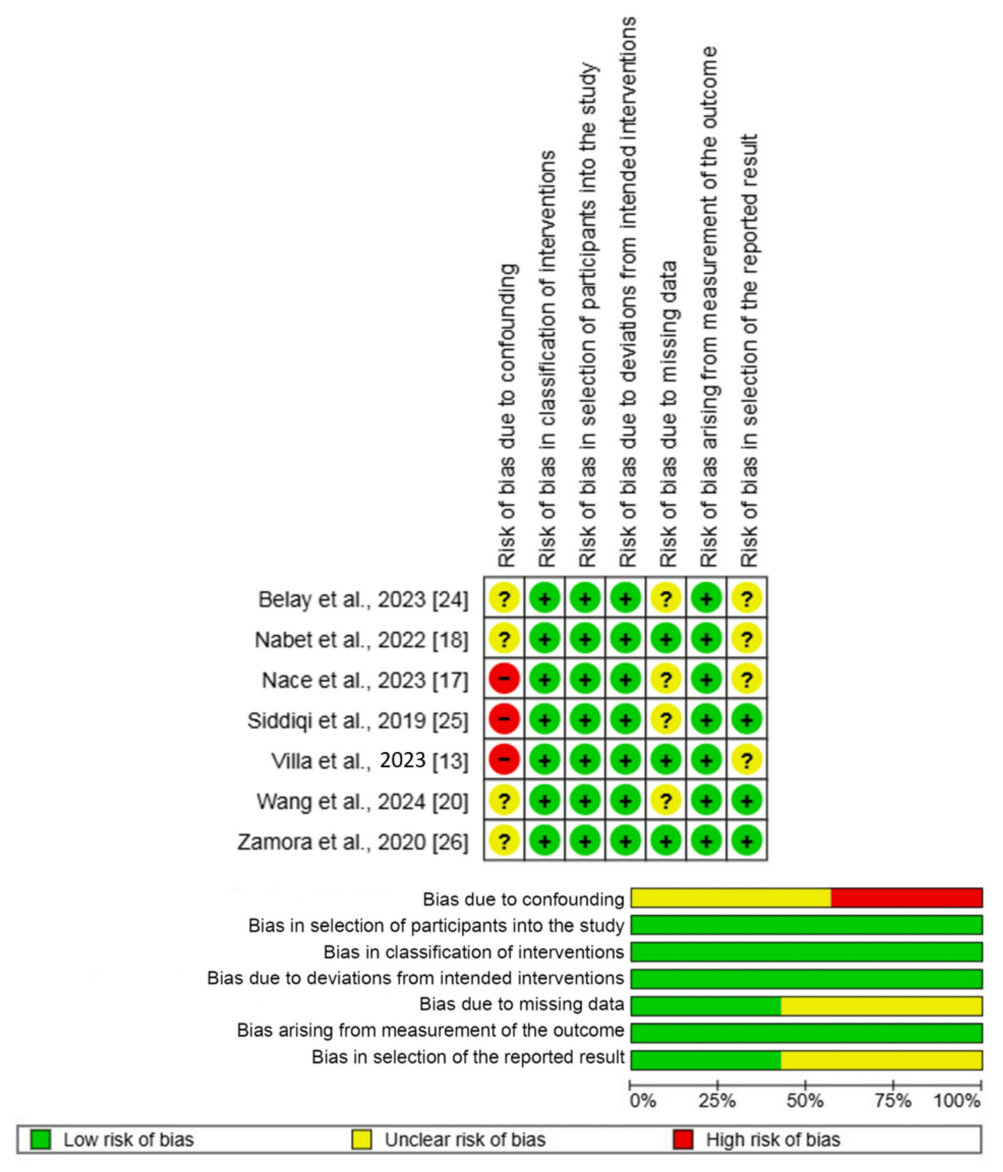
Risk of bias summary and graph showing authors' judgments about each risk of bias item for non-randomized observational studies References: [13,17,18,20,24-26]

Figure 2 outlines the outcome of the risk of bias assessment of the included studies. A serious risk of bias was identified in three studies for the overall assessment, which was driven by a high risk of bias due to confounding in those same studies [13,17,25]. The risk of bias due to selection of the reported result was judged to be moderate in six studies. For all other domains, the risk of bias was judged to be either low or moderate across the included studies.

Outcome synthesis

Reinfection

**Figure 3:**
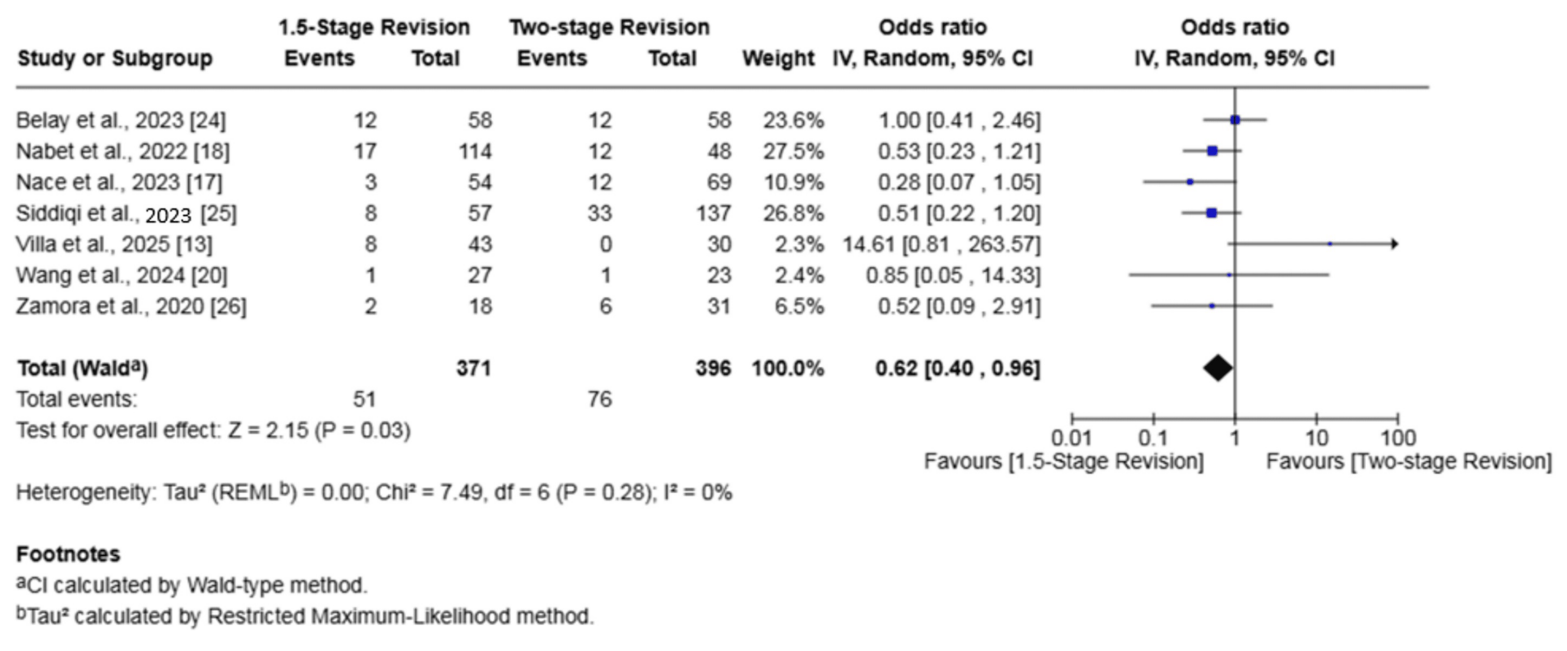
Forest plot of comparison of reinfection in patients undergoing 1.5-stage versus two-stage revision for prosthetic joint infection. The solid squares denote the odds ratio (OR). The horizontal lines represent the 95% confidence intervals (CIs), and the diamond denotes the pooled effect size. References: [13,17,18,20,24-26]

Seven studies (767 patients) reported on reinfection [13,17,18,20,24-26]. The rate of reinfection in the 1.5-stage revision group was 13.75% whilst it was 19.19% in the two-stage revision group. The 1.5-stage revision was associated with a significantly lower risk of reinfection (OR 0.62, 95% CI 0.40-0.96, p = 0.03). No heterogeneity was found among the evaluated studies (I² = 0%, p = 0.28) (Figure 3).

Failure of Infection Eradication

**Figure 4:**
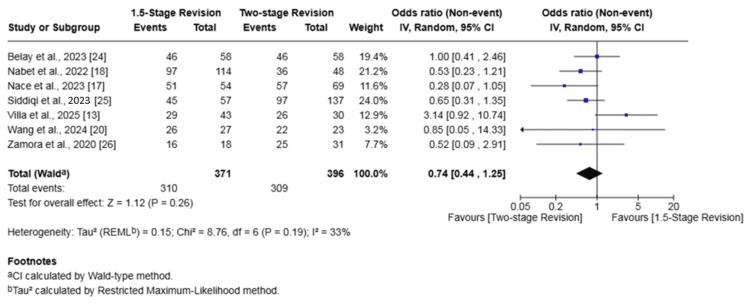
Forest plot of comparison of failure of infection eradication in patients undergoing 1.5-stage versus two-stage revision for prosthetic joint infection. The solid squares denote the odds ratio (OR). The horizontal lines represent the 95% confidence intervals (CIs), and the diamond denotes the pooled effect size References: [13,17,18,20,24-26]

Seven studies (767 patients) investigated the failure of infection eradication [13,17,18,20,24-26]. The rate of failure in the 1.5-stage revision group was 16.4% versus 22.0% in the two-stage revision group. There was no significant difference in the odds of failure of infection eradication between the two groups (OR 0.74, 95% CI 0.44-1.25, p = 0.26). There was low heterogeneity among studies (I2 = 33%, p = 0.19) (Figure 4).

Aseptic Loosening

**Figure 5:**
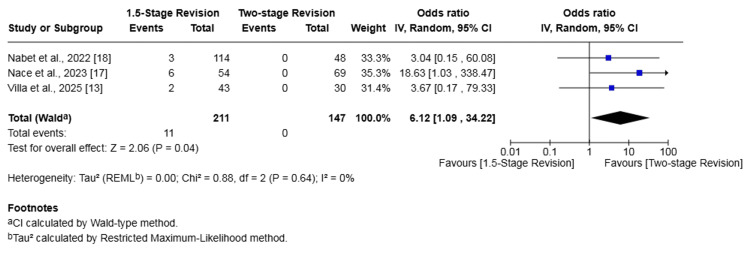
Forest plot of comparison of aseptic loosening in patients undergoing 1.5-stage versus two-stage revision for prosthetic joint infection. The solid squares denote the odds ratio (OR). The horizontal lines represent the 95% confidence intervals (CIs), and the diamond denotes the pooled effect size References: [13,17,18]

Three studies (358 patients) investigated aseptic loosening [13,17,18]. The rate of aseptic loosening in the 1.5-stage revision group was 5.21% versus 0.00% in the two-stage revision group. The 1.5-stage revision was associated with a significantly higher risk of aseptic loosening (OR 6.12, 95% CI 1.09-34.22, p = 0.04). There was no heterogeneity among studies (I² = 0%, p = 0.64) (Figure 5).

Overall Complications

**Figure 6:**
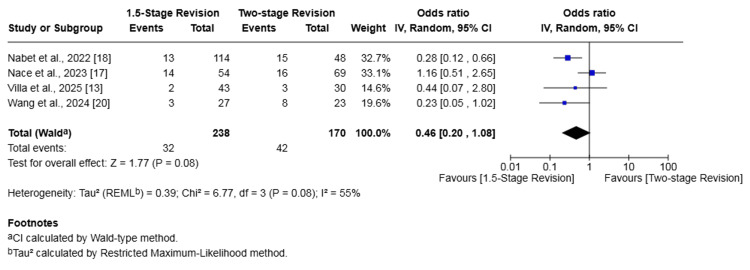
Forest plot of comparison of overall complications in patients undergoing 1.5-stage versus two-stage revision for prosthetic joint infection. The solid squares denote the odds ratio (OR). The horizontal lines represent the 95% confidence intervals (CIs), and the diamond denotes the pooled effect size References: [13,17,18,20]

Four studies (408 patients) reported on overall complications [13,17,18,20]. The rate of complications was 13.45% in the 1.5-stage revision group and 24.71% in the two-stage revision group. The difference in risk was not statistically significant (OR 0.46, 95% CI 0.20-1.08, p = 0.08). Heterogeneity was moderate (I² = 55%, p = 0.08) (Figure 6). A detailed breakdown of the specific complications reported in these four studies is provided in Table 3.

**Table 3:** Complication breakdown ¹'Other' for Nabet et al., 2022 [18] includes cellulitis, wound dehiscence, acute kidney injury, and amputation MUA: manipulation under anaesthesia; PE: pulmonary embolism; DVT: deep vein thrombosis References: [13,17,18,20,24-26]

Study (joint)	Complication type	1.5-stage group (events/total)	Two-stage group (events/total)
Belay et al., 2023 [24]	Complications	NR	NR
Nabet et al., 2022 [18]	Aseptic loosening	3/114	0/48
	Dislocation	1/114	0/48
	Periprosthetic fracture	2/114	1/48
	Stiffness (lysis/MUA)	4/114	10/48
	Other¹	3/114	4/48
Nace et al., 2023 [17]	Aseptic loosening	6/54	0/69
	Dislocation	0/54	3/69
	Periprosthetic fracture	2/54	0/69
	Pain/gait dysfunction	1/54	1/69
	PE/DVT	1/54	0/69
Siddiqi et al., 2023 [25]	Complications	NR	NR
Villa et al., 2025 [13]	Aseptic loosening	2/43	0/30
	Periprosthetic fracture	0/43	1/30
	Instability	0/43	1/30
	Sciatic nerve palsy	0/43	1/30
Wang et al., 2024 [20]	Spacer fracture	0/27	5/23
	Spacer dislocation	2/27	2/23
	Periprosthetic fracture	1/27	1/23
Zamora et al., 2020 [26]	All complications	NR	NR

Readmission

**Figure 7:**
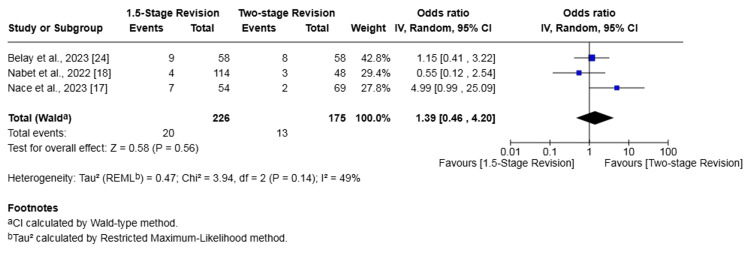
Forest plot of comparison of readmission in patients undergoing 1.5-stage versus two-stage revision for prosthetic joint infection. The solid squares denote the odds ratio (OR). The horizontal lines represent the 95% confidence intervals (CIs), and the diamond denotes the pooled effect size References: [17,18,24]

Three studies (401 patients) reported on readmission [17,18,24]. The rate of readmission in the 1.5-stage revision group was 8.85% whilst it was 7.43% in the two-stage revision group. This difference was not statistically significant (OR 1.39, 95% CI 0.46-4.20, p = 0.56). There was moderate heterogeneity among studies (I² = 49%, p = 0.14) (Figure 7).

Periprosthetic Fracture

**Figure 8:**
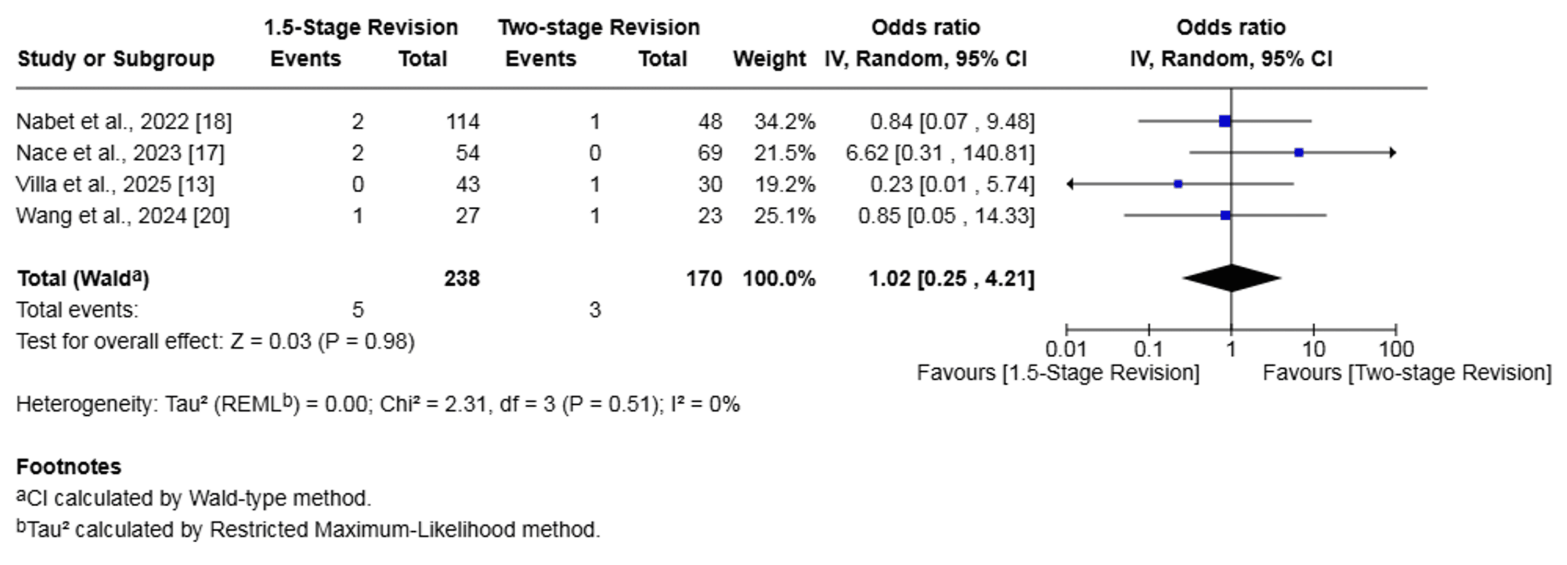
Forest plot of comparison of periprosthetic fracture in patients undergoing 1.5-stage versus two-stage revision for prosthetic joint infection. The solid squares denote the odds ratio (OR). The horizontal lines represent the 95% confidence intervals (CIs), and the diamond denotes the pooled effect size References: [13,17,18,20]

Four studies (408 patients) reported on periprosthetic fracture [13,17,18,20]. The rate was 2.10% in the 1.5-stage revision group and 1.76% in the two-stage revision group. The difference in risk was not statistically significant (OR 1.02, 95% CI 0.25-4.21, p = 0.98). No heterogeneity was found (I² = 0%, p = 0.51) (Figure 8).

Dislocations

**Figure 9:**
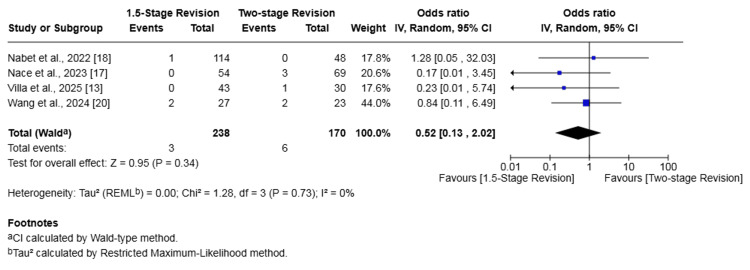
Forest plot of comparison of dislocation in patients undergoing 1.5-stage versus two-stage revision for prosthetic joint infection. The solid squares denote the odds ratio (OR). The horizontal lines represent the 95% confidence intervals (CIs), and the diamond denotes the pooled effect size References: [13,17,18,20]

Four studies (408 patients) reported on dislocations [13,17,18,20]. The rate of dislocation in the 1.5-stage revision group was 1.26% whilst it was 3.53% in the two-stage revision group. The difference in risk was not statistically significant (OR 0.52, 95% CI 0.13-2.02, p = 0.34). No heterogeneity was found among the evaluated studies (I² = 0%, p = 0.73) (Figure 9).

Subgroup analysis

Hip PJI

**Figure 10:**
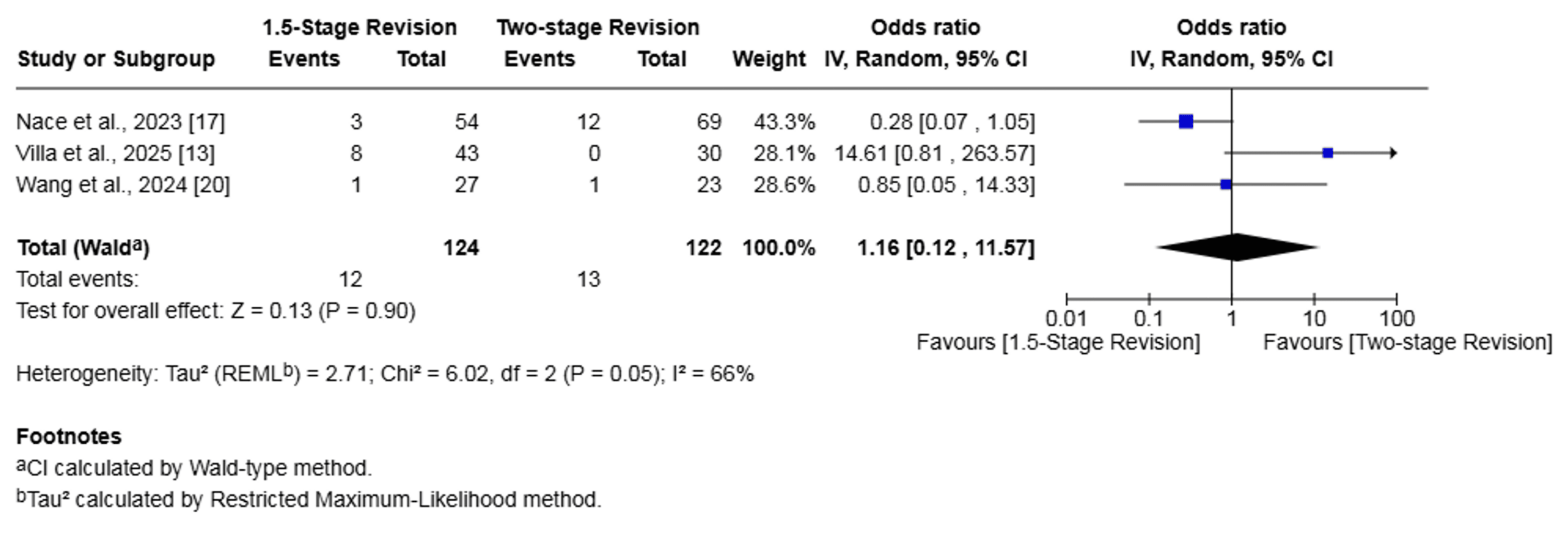
Subgroup forest plot of comparison of reinfection in patients undergoing 1.5-stage versus two-stage revision for hip periprosthetic joint infection. The solid squares denote the odds ratio (OR). The horizontal lines represent the 95% confidence intervals (CIs), and the diamond denotes the pooled effect size References: [13,17,20]

**Figure 11:**
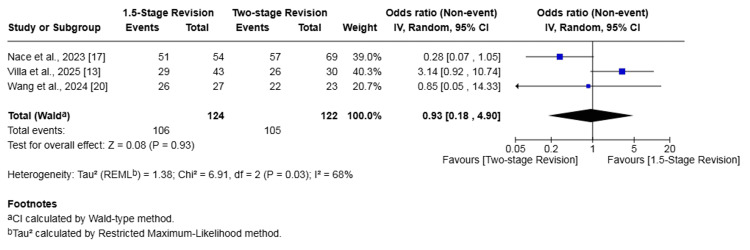
Subgroup forest plot of comparison of failure of infection eradication in patients undergoing 1.5-stage versus two-stage revision for hip periprosthetic joint infection. The solid squares denote the odds ratio (OR). The horizontal lines represent the 95% confidence intervals (CIs), and the diamond denotes the pooled effect size References: [13,17,20]

Subgroup analysis of three studies (246 patients) [13,17,20] reporting on outcomes for hip PJI demonstrated no significant difference between the 1.5-stage and two-stage revision groups for either reinfection or failure of infection eradication. Specifically, the reinfection rate in the 1.5-stage revision group was 9.68% compared to 10.66% in the two-stage group, a difference that was not statistically significant (OR 1.16, 95% CI 0.12-11.57, p = 0.90). Similarly, the infection eradication failure rate was 14.52% in the 1.5-stage group versus 13.93% in the two-stage group, which was also not a significant difference (OR 0.93, 95% CI 0.18-4.90, p = 0.93). Substantial heterogeneity was detected for both outcomes (I² = 66% and I² = 68%, respectively) (Figures 10, 11).

Knee PJI

Subgroup analysis of four studies (521 patients) [18,24-26] for knee PJI demonstrated a statistically significant lower rate of reinfection with the 1.5-stage revision. There was no significant difference in the rate of failure of infection eradication between the two groups.

**Figure 12:**
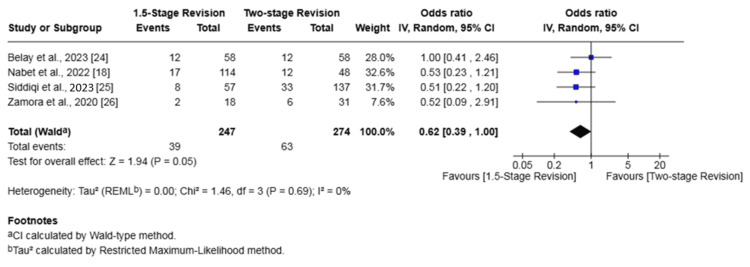
Subgroup forest plot of comparison of reinfection in patients undergoing 1.5-stage versus two-stage revision for knee periprosthetic joint infection. The solid squares denote the odds ratio (OR). The horizontal lines represent the 95% confidence intervals (CIs), and the diamond denotes the pooled effect size References: [18,24-26]

**Figure 13:**
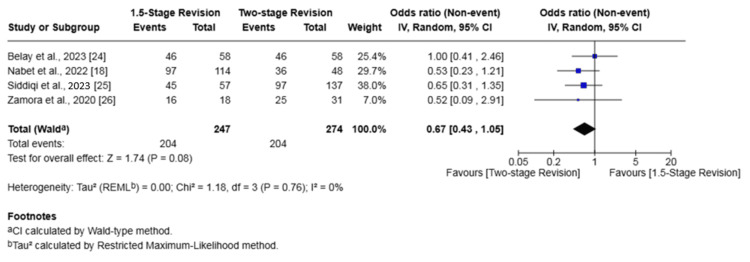
Subgroup forest plot of comparison of failure of infection eradication in patients undergoing 1.5-stage versus two-stage revision for knee periprosthetic joint infection. The solid squares denote the odds ratio (OR). The horizontal lines represent the 95% confidence intervals (CIs), and the diamond denotes the pooled effect size References: [18,24-26]

The analysis showed a reinfection rate of 15.79% in the 1.5-stage revision group versus 22.99% in the two-stage revision group (OR 0.62, 95% CI 0.39-1.00, p = 0.05). For infection eradication, the failure rate was 17.41% in the 1.5-stage group compared to 25.55% in the two-stage group, which was not a statistically significant difference (OR 0.67, 95% CI 0.43-1.05, p = 0.08). No heterogeneity was found among the studies for either outcome (I² = 0%) (Figures 12, 13).

Sensitivity Analysis

The direction of the pooled effect was unchanged whether expressed as a risk ratio (RR) or risk difference (RD). During the leave-one-out sensitivity analysis, in the analysis of failure of infection eradication, removal of the study by Villa et al. [13] changed the pooled effect to an OR of 0.61 (95% CI 0.40-0.94; p = 0.02), favouring the 1.5-stage revision, with zero heterogeneity (I² = 0%). In the analysis of complications, removal of the study by Nace et al. [17] made the results significant (p = 0.0003), favouring the 1.5-stage revision, with zero heterogeneity (I² = 0%).

Discussion

This meta-analysis has shown that reinfection rates for overall PJIs are significantly lower for 1.5-stage procedures compared to two-stage procedures. However, aseptic loosening was higher in the 1.5-stage group. Our subgroup analysis looking at THA and TKA separately did not show any difference; however, there was a trend towards lower reinfection rates and higher infection eradication in TKA PJI managed with a 1.5-stage procedure. The findings highlight the growing attraction for 1.5-stage revision; however, patient selection is critical. There is, however, a need for higher-quality studies that also consider patient-reported outcomes.

We found a statistically significant reduction in overall reinfection rate and a trend towards lower failure of infection eradication for periprosthetic hip and knee joint infections. These results support the use of 1.5-stage procedures, as in addition to infection eradication and reinfection benefits, the 1.5-stage procedure is more cost-effective and has reduced psychological burden [16,25,27]. The reduced psychological burden can support faster recovery and return to function [25]. Patient selection for a 1.5-stage procedure is critical, and patients with significant bone loss or poor soft tissue envelopes would not be good candidates [25]. For TKA, patients with deficient collateral ligaments resulting in an unstable knee would also not benefit from a 1.5-stage procedure [28].

However, it is worth noting the work of Villa et al., who found that patients undergoing a 1.5-stage stem hybrid fixation had a significantly higher rate of re-revisions due to infection (p = 0.011), with 80% of failures in that group attributed to uncontrolled infection [13]. The authors presented a novel modification by using a cementless implant with cement between the splines. This method potentially reduces the antibiotic load available through the reduced amount of cement. In addition, the groups in this study were not matched with the 1.5-stage group having higher American Society of Anaesthesiologists (ASA) grades and more prior revisions [13]. Our leave-one-out sensitivity analysis should significantly lower the failure of infection eradication rates (OR 0.61 (95% CI 0.40-0.94; p = 0.02)) when the results of Villa et al. were excluded.

Conversely, our analysis also revealed that the 1.5-stage revision was associated with a significantly higher risk of aseptic loosening. This finding is supported by the radiographic review from Nace et al., which reported more patients without progressive radiolucency in the two-stage cohort. Specifically, 94% of two-stage recipients showed no femoral radiolucency, and 90% showed no acetabular radiolucency, whereas this was true for only 82% of patients in the 1.5-stage group [17]. A potential mechanism for this is that the 1.5-stage revision may fail secondary to aseptic loosening due to the addition of high-dose antibiotics to the cement, which could compromise its long-term mechanical integrity [16]. Not all authors described the cementing technique, and therefore, it is possible that if a third-generation cementing technique was not used, then this can contribute to higher aseptic loosening rates [29]. The mean follow-up in our study was 36.0 months, and therefore, the true rate of aseptic loosening could be higher with a 1.5-stage revision.

Although two-stage revision has traditionally been considered the gold standard for treating PJI [25,30], the approach is not without considerable drawbacks. It significantly reduces patient activity time, and as noted by Zhao et al., the removal of a well-immobilized prosthesis may lead to the degeneration of bone stock and increase the risk of perioperative fractures [31]. This study, however, did not identify a difference between the periprosthetic fracture rates between the two groups. The complication rate was lower in the 1.5-stage group (13.45% vs. 24.71%), although the difference was not statistically significant. This trend was expected as recurrent operations increase the risk of complications, and spacers used in two-stage procedures are associated with dislocations and fractures [32].

It is important to note that almost all included studies in this meta-analysis originated from North America (five papers from the United States and one from Canada), with only one paper from China. This geographic concentration could impact our findings. It is established that the profile of infecting organisms and their antimicrobial resistance patterns can vary between different global populations [33,34]. A treatment protocol's success, which is heavily dependent on antibiotic efficacy, may therefore differ in settings outside of North America. Future, larger-scale international studies are needed to validate these results across diverse patient populations and healthcare environments. In addition, future studies should focus on developing risk prediction models to identify patients who are most likely to succeed with a 1.5-stage revision. A strong indication for 1.5-stage revision previously has been for patients with multiple comorbidities who are unlikely to do well with two operations; however, patients who have fewer comorbidities may also benefit from a 1.5-stage procedure.

The current study has some limitations that should be considered when interpreting its findings. All of the included studies were retrospective cohort studies, which are subject to selection bias. This could be influenced by patients with adequate functionality being more likely to prefer the retention of their articulating spacer than those with unsatisfactory results [26]. Although a high-quality prospective randomized controlled trial would be necessary to definitively recommend one treatment strategy, longer-term prospective studies are warranted to better evaluate these outcomes. Furthermore, many of the included reports were single-centre studies with small sample sizes, which may lack the statistical power to detect some differences and increase the risk of a type 2 error. The generalizability of the findings may also be limited, as it is unknown whether these results can be applied to geographic regions with different microorganism prevalence. We were not able to evaluate the outcomes with respect to specific subgroups, such as culture-negative versus culture-positive PJI, organism type, or high-risk patients. Finally, we did not evaluate long-term outcomes such as the rate of aseptic failures or five-year survival with infection clearance.

## Conclusions

The meta-analysis of best available evidence (Level 2a) indicates that a 1.5-stage revision for PJI is associated with a significantly lower rate of reinfection, but a higher risk of aseptic loosening compared to the two-stage approach. This trade-off between infection control and mechanical durability, identified from observational data, underscores the need for higher-level evidence. Future high-quality randomized controlled trials are necessary to definitively establish the optimal surgical strategy.

## Appendices

**Table 4:** Search strategy *This search strategy was adopted for the following databases: MEDLINE, EMBASE, CINAHL, Cochrane Central Register of Controlled Trials (CENTRAL), and Web of Science

Search No	Search strategy^*^
#1	MeSH descriptor: [ Arthroplasty, Replacement, Revision ] explode all trees
#2	"""1.5-stage"" OR ""one and a half stage"" OR ""2-stage"" OR ""two stage"" OR ""staged revision"" : TI,AB,KW"
#3	#1 OR #2
#4	MeSH descriptor: [ Prosthesis-Related Infections ] explode all trees
#5	"""periprosthetic joint infection"" OR ""PJI"" OR ""prosthetic joint infection"" OR ""infected arthroplasty"" : TI,AB,KW"
#6	#4 OR #5
#7	MeSH descriptor: [ Postoperative Complications ] explode all trees
#8	"""outcomes"" OR ""complications"" OR ""reinfection"" OR ""eradication"" OR ""failure"" OR ""adverse events"" : TI,AB,KW"
#9	#7 OR #8
#10	MeSH descriptor: [ Arthroplasty, Replacement ] explode all trees
#11	"""arthroplasty"" OR ""joint replacement"" OR ""total joint arthroplasty"" OR ""TJA"" : TI,AB,KW"
#12	#10 OR #11
#13	#3 AND (#6 OR #9) AND #12
